# Optimization of the Electrochemical Method of Obtaining Graphene Nanoplatelets (GNPs)

**DOI:** 10.3390/ma16062188

**Published:** 2023-03-09

**Authors:** Adrianna Grabowska, Jerzy Kowalczyk, Robert Tomala, Maciej Ptak, Małgorzata Małecka, Anna Wędzyńska, Mariusz Stefanski, Wiesław Stręk, Paweł Głuchowski

**Affiliations:** 1 Faculty of Chemistry, Wroclaw University of Science and Technology, 50-370 Wroclaw, Poland; 2 Institute of Low Temperature and Structure Research, Polish Academy of Sciences, 50-422 Wroclaw, Poland

**Keywords:** graphene nanoplatelets, electrolysis, exfoliation in liquid-phase, Raman spectroscopy, IR, XRD, TEM, zeta potential

## Abstract

Graphene nanoplatelets (GNPs) were prepared using the electrolytic exfoliation method on graphite foil in an ammonium sulfate solution. A series of experiments were conducted in order to optimize the production of the flakes by varying the pH of the solution, applied voltage and current, duration of electrolysis, temperature in the electrolytic system, and type and duration of the ultrasound interaction. The quality of the produced graphene nanoplatelets was analyzed using X-ray diffraction, Raman and IR spectroscopy, and TEM.

## 1. Introduction

Graphene is a two-dimensional (2D) material that is one atom thick and was first isolated by Geim and Novoselov in 2004 [1]. Since then, it has become one of the most researched materials thanks to its unique properties. Its structure contains carbon atoms bonded together in a hexagonal pattern, similar to that of a honeycomb. Graphene is the building block for all forms of graphite [2]. The carbon atoms in the monolayer are connected to each other by strong bonds with sp^2^ hybridization. These σ bonds are responsible for the high mechanical strength of the material [3]. It is worth mentioning that the number of carbon layers is crucial in determining the quality of the product. Carbon materials formed by more than one but not more than 10 carbon layers are considered graphene, while this number ranges from 10 to 100 for graphene nanoplatelets (GNPs) [4]. The overlapping monolayers are connected by weak π bonds. These relatively weak bonds give rise to the exceptional optical, electrical, and thermal properties of graphene [5,6,7]. The uniqueness of graphene is confirmed by the ability of a single layer to absorb only about 2.3% of white light, which results in its high transparency [5]. A membrane made from it is impermeable to gases, while also not impeding water [8,9]. Another amazing attribute of graphene is its high tensile strength of 130 GPa, which is more than 200 times higher than steel of equal mass (0.376 GPa) [10]. In addition, it can also be stretched up to as much as 20% without damage; its elastic limit and Young’s modulus are 1 and 0.5 TPa, respectively [11]. The electrical conductivity of graphene can significantly exceed the values achieved at room temperature in good metallic conductors; accordingly, graphene is considerable as an ideal conductor [6,12].

Graphene can be prepared in various ways, differing in price, ease of fabrication, and quality of the obtained product. The most common synthesis methods include chemical vapor deposition (CVD) [13,14,15], mechanical exfoliation [16,17], graphene oxide reduction [18,19], liquid-phase exfoliation [20,21], and epitaxial growth on silicon carbide [22]. Among the mentioned techniques, the graphite exfoliation method is ideal for applications requiring the production of industrial amounts of graphene [23]. Exfoliation of graphite in the liquid phase can be achieved using various techniques. One of the easiest and most accessible ways is to exfoliate using ultrasound together with substances that minimize the surface tension at the liquid–graphene interface (e.g., *N*,*N*-dimethylformamide (DMF)) [24]. Another method, which is commonly referred to as chemical exfoliation was developed by Viculis et al. in 2005 [25]. The scientists used a simple intercalation and exfoliation procedure. The graphite was intercalated with potassium salts, which led to the formation of KC_8_. The KC_8_ was then reacted with ethanol in order to form potassium ethanolate, thereby causing exfoliation and resulting in the formation of a carbon monolayers. The byproduct of this process was hydrogen, which helped to separate the single graphene layers from each other. A similar method based on the switching voltage technique and eutectic molten salts of NaOH and KOH was applied for graphite exfoliation by Alshamkhani et al. [26]. The third technique is an electrochemical method based on the usage of a liquid electrolyte and direct current [27]. Compared to other concepts, the electrochemical method of graphene fabrication offers a number of advantages, such as ease of operation, low fabrication temperature, easy control of the process by changing the electrolysis parameters, environmental friendliness, and significant synthesis rates [28,29]. This process is based on electrolysis during which the anode is graphite, while the choice of cathode is arbitrary. Under the influence of an electric field, the graphite electrode is exfoliated by gas bubbles released from the solution in which they are immersed. By applying a negative potential to the graphite electrode, excessive oxidation can be prevented, thus protecting the graphite structure from deformation due to the attachment of functional groups. The selection of the electrolyte is of crucial importance in this process. Therefore, there have been numerous reports in the literature on both experiments with the use of aqueous and nonaqueous electrolytes [30]. The advantage of nonaqueous solutions is their wide electrochemical windows, which allow for a more stable process. However, their main disadvantages are the extended electrolysis time and toxic byproducts. Electrolytes formed by acids or inorganic salts are also used in cases where graphite serves as an anode [31]. On the other hand, water is an attractive solvent due to its availability and sustainable nature. It not only acts as a solvent, but also creates bonds between carbon and oxygen in the initial stage of electrolysis. The negatively charged anions cause the oxygen to be pushed out in molecular form, initiating further exfoliation. It was revealed that, among many electrolyte solutions, sulfuric acid(VI) was the most promising due to the rapid reduction of sulfate ions, allowing for the detachment of carbon monolayers [32,33]. The disadvantage of this solution is excessive oxidation and formation of graphene oxide (GO) with a defective surface. Therefore, sulfuric acid was replaced by salts containing SO_4_^2−^ anions [34,35].

In the presented study, a combination of two types of liquid-phase exfoliation was used to produce graphene: electrochemical and ultrasonic extraction. In order to obtain a product of the best possible quality, parameters such as process duration, applied current and voltage, pH of the solution, time and type of the ultrasound interaction, and electrolyte temperature were modified. To the best of our knowledge, this is the first paper where systematic research has been undertaken to define the influence of the mentioned factors on the quality and quantity of the obtained graphene during electrolysis process.

## 2. Materials and Methods

Ammonium sulfate ((NH_4_)_2_SO_4_, Sigma Aldrich, ≥99.0%), pure graphite blocks, and graphite foils (GFC99M1—0.1 cm thick and GFC99M2—0.2 cm thick, Sinograf Toruń S.A.) were used as starting materials. In order to change the pH of the electrolyte, nitric acid (HNO_3,_ POCH, analytical grade, 65%), ammonia solution (NH_3_·H_2_O, POCH, 25%), and sodium hydroxide (NaOH, POCH, ≥98.8%) were used. The solutions were made with the use of distilled water with a conductivity of 0.51 μS/cm and pH = 7. The graphene nanoplatelet reference material was produced by ACS MATERIAL (Graphene Nanoplatelets, 1–2 nm thickness, SKU: GNNP01A5). Aqueous solutions of acetone (POCH, analytical grade, 99.5%) and ethanol (POCH, analytical grade, 96%) were used in the ultrasonification stage. Hard filters type 380 with pore sizes in the range of 3–5 µm were used for filtration.

In the electrolysis process, a laboratory power supply from STAMOS (Model: S-LS-38) with a voltage regulated in the 0–30 V range and a current regulated in the 0–20 A was used to adjust the current flow through the electrolytic system. Liquid-phase exfoliation was obtained by the TF-900N sonicator by Tefic Biotech and the Proclean 3.0DSP ultrasonic cleaner by Ulsonix. XRD measurements were carried out on an X-ray diffractometer (XRD, X’Pert PRO, PANalytical, Bruker, Germany) with Cu Kα monochromatic radiation (λ = 1.5406 Å), scanning the diffraction angles (2θ) between 10° and 55° at room temperature in Bragg–Brentano geometry. The measurements of Raman spectra were performed with a Renishaw inVia Microscope Raman spectrophotometer equipped with a CCD detector. The tests were performed at room temperature using the 514 nm line of the argon laser. The spectral range was 200–3250 cm^−1^, with a resolution of up to 0.1 cm^−1^. IR spectra in the range of 4000–400 cm^−1^ (mid-IR) were measured using a Nicolet iS50 infrared spectrometer using a KBr pellet method. The spectral resolution was 2 cm^−1^. The morphology of the flakes was studied using a Philips CM-20 SuperTwin transmission electron microscope with an accelerating voltage of 200 kV and a resolution of 0.24 nm. Scanning electron microscopy (SEM) FEI NovaNanoSEM 230 (USA) was used to reveal the graphene nanoplatelet thickness obtained at different voltages. The oxygen content in the GNPs was measured using an FESEM FEI Nova NanoSEM 230 scanning electron microscope equipped with an EDS spectrometer (EDAX Genesis). The zeta potential of the aqueous suspension of graphene nanoplatelets was measured and analyzed under the same conditions (same weight of graphene and volume of water; same time of ultrasound dispersion) using the a Zetasizer Nano ZS (Malvern Instruments, Malvern, Worcestershire, UK). The materials were dried in a FD-10-R Vacuum Freeze-Dryer from LABFREEZ (Beijing, China).

**Figure 1:**
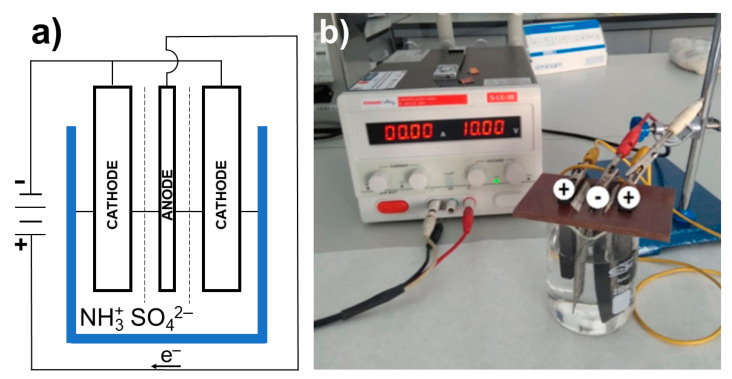
Experimental setup used for electrochemical exfoliations: (**a**) scheme; (**b**) photograph.

The electrolytic system consisted of two cathodes, being two blocks of pure graphite with dimensions of 7 cm × 2 cm × 1 cm, and one anode, which was cut out of graphite foil into dimensions of 9 cm × 2 cm × 0.1/0.2 cm. The electrodes were 2 cm apart from each other and separated by spacers to counteract electrophoresis in the solution (Figure 1). Electrochemical exfoliation was obtained using two cathodes in order to maintain a constant current flow. The electrolysis process was performed in 225 mL of 0.1 M aqueous solution of ammonium sulfate. Attempts were also made to replace the water in the electrolyte with 50% and 33% acetone solutions. The use of a 50% solution was found to have adverse effects on the quantity of the product. The results for the samples obtained with a 25% acetone solution are presented in Section 3. Each electrolysis started with activation of the electrodes by applying 2.5 V voltage for 10 min. The next step was to carry out the proper electrolysis at a constant voltage and constant external conditions. After the electrolysis, the obtained precipitate was filtered off, washed with distilled water, and divided into equal parts, which were used to prepare aqueous or acetone solutions. The solutions were subjected to ultrasound of different power and for different periods of time.

The electrolysis process in an appropriately selected aqueous electrolyte proceeded as follows: hydroxyl and oxygen radicals were formed at the electrode during electrolysis of water. The resulting radicals initially oxidized the edges of the graphite grains, which led to depolarization and expansion of the graphite layers, thus facilitating intercalation of SO_4_^2−^ ions. The next step was the reduction of intercalated SO_4_^2−^ and the self-oxidation of water, the products of which were gas bubbles (SO_2_ and O_2_). The released gas destroyed the weak bonds, detaching graphite layers one from another. During the electrolysis process, it was possible to observe the release of hydrogen at the cathode and oxygen at the anode. Sediment accumulated at the bottom and part of the exfoliated material floated on the surface of the solution. The current increased during the process due to acidification of the electrolyte and the increase in graphene content in the solution. In order to obtain optimal electrolysis conditions, the following parameters were modified: voltage (from 6 V to 12 V), temperature of electrolyte (from 5 to 25 °C), time of the process (from 45 min to 90 min), and pH (from 3.0 to 10.3) of the electrolyte (Table 1). The suspension was subjected to ultrasound with a frequency of 50 Hz and variable power. The samples in the sonicator were subjected to a series of 3 s pulses interrupted by 3 s pauses at 230 or 630 W of power, with time varied from 15 to 90 min. The samples during exfoliation under ultrasound were subjected to different temperatures. An attempt was also made to replace distilled water with a 75% acetone solution and a 66% ethanol solution; however, due to the low freezing point of solution of ethyl alcohol, equal to −114 °C, it was impossible to freeze the solution, which was crucial to obtain a dry product by sublimation process. For this reason, further studies on the effect of ethanol were abandoned. For each electrolysis, a reference sample was prepared, which was not sonicated. After 72 h, the supernatants were collected and dried in a vacuum freeze-dryer to produce a foam resembling black powder.

## 3. Results

### 3.1. XRD Analysis

**Figure 2:**
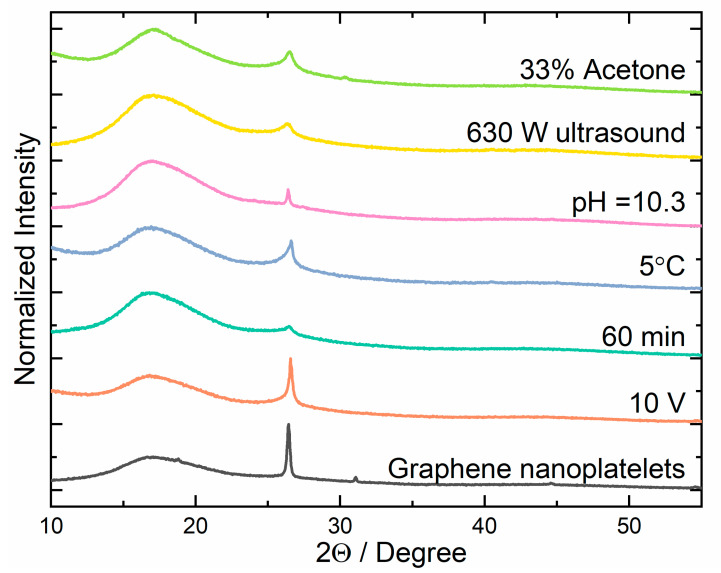
XRD patterns of the selected samples after optimization of synthesis parameters (electrochemical exfoliation with a 10 V voltage, after 60 min of the process; synthesis in the solution chilled to 5 °C, after processing in a solution with an alkaline pH = 10.3, after sonification of the product with 630 W ultrasound, and after sonification of the GNPs in a solution of 67% water and 33% acetone), along with the reference sample.

In order to investigate the purity of the prepared graphene nanoplatelets, X-ray diffraction analysis was performed. The results obtained for the GNPs were compared to those obtained for commercial samples and are presented in Figure 2. The diffractograms contain a characteristic broad band from 10° to 23° and a peak at 27° corresponding to graphene and graphite, structures, respectively [36,37,38]. The first is associated with the reflection of radiation between the sp^2^ planes, while the second one is due to the crystalline structure of graphite. The different shapes of the peaks are caused by differences in the structure of the investigated materials. Several parameters have been changed in order to optimize the electrolysis process and to obtain the best quality product (highest graphene content, lowest graphene oxide and graphite impurities, and low defect number). The XRD results of the samples with the highest efficiency of graphene to graphite formation for a particular factor are presented in Figure 2. The diffractograms obtained for samples prepared under different conditions are shown in Supplementary Figures S1–S7.

To be able to compare results for samples obtained under different conditions, the content of graphene in the final product was determined on the basis of the XRD measurements. The area of the peak assigned to the graphite and a broad band related to the reflections of graphene sp^2^ planes were used for the calculations. The ratio of the area under the graphene band and the graphite peak allows determining their share in the particular sample [39]. The graphene content (C) was determined according to the following formula:

(1) $$C=\frac{\int_{10}^{23}f(x)}{\int_{10}^{23}f(x)+\int_{23}^{32}g(x)}\times 100\%,$$

where $\int_{10}^{23}f(x)$ is the integral of the area under the band corresponding to graphene, and $\int_{23}^{32}g(x)$ is the integral of the surface under the peak corresponding to the graphite structure. The graphene content (*C*) calculated for all samples is presented in Supplementary Figures S1–S7.

The first variable that was checked was the voltage of the current flowing through the circuit. To optimize it, the experiment was conducted under several voltages: 6 V, 8 V, 10 V, and 12 V. XRD analysis indicated that the purity of the obtained graphene nanoplatelets was comparable for the three highest applied voltages and resulted in the formation of about 90% of the graphene content in the samples. Just the application of the lowest voltage resulted in strong product contamination (Figure S1). The reason for such behavior may be an insufficient potential to reduce SO_4_^2−^ to SO_2_ and a consequent absence of bubbles that exfoliate the graphene flakes. Another factor, the duration of the electrolysis process, was determined experimentally by running the process for the period of 45, 60, and 90 min. The analysis of the diffraction patterns showed that the highest graphene content in the final product was obtained for the 60 min process (Figure S2). This shows that, if a shorter time period is used, a part of the graphite that is in sediment remains still not exfoliated; if the duration is too long, the foil used as an anode starts to be too thin, and, instead of the foil exfoliation, particles of graphite fall off. This shows just how important it is to observe reaction and stop it at the appropriate stage. The next factor whose impact on the reaction efficiency and quality of the obtained graphene was investigated was the temperature of the electrolyte during the exfoliation process. It was found that reaction temperature had a significant influence on both the quantity and the purity of the obtained product. Lowering the process temperature from 25 °C to 5 °C led to a doubling of the amount of graphene in the solution. It is well known that the current flowing through the electrodes increases the temperature of the electrolyte. Placing a reaction container at the lower temperature helps to prevent heating of the reaction environment and increases the exfoliation efficiency. The lower temperature of the solution prevents the graphene nanoplatelets from sticking together. XRD diffractograms of GNPs obtained at various temperatures are presented in Figure S3. The pH of 0.1 M NH_4_(SO_4_)_2_ standard solution used as the electrolyte in the exfoliation process was 5.3. To check the impact of pH on the course of reaction, the electrolyte was modified using nitric acid (HNO_3_) and ammonia water (NH_3_·H_2_O). The XRD analysis showed that an increase in the pH of the electrolyte had a positive effect. The content of graphene in the obtained product was increased at higher pH values (Figure S4). This may be related to the fact that, at lower pH, fewer SO_4_^2−^ ions were reduced to SO_2_, and the resulting gas did not detach graphene nanoplatelets from the graphite foil.

The next step in optimization of the electrolysis process was to check the impact of postprocessing of the solution obtained during electrolysis using ultrasound. It was found that the use of ultrasound had a large positive impact on the quality of the obtained product. The first step in optimization of the ultrasound usage was the selection of the optimal type and power. For this purpose, the effect of 230 W continuous ultrasound was compared with pulsating 3 s cycles of ultrasound with a power of 630 W. Analyzing the XRD patterns, it can be noticed that, under sonification, the quality of graphene strongly increased (Figure S5) and was comparable for low- and high-power ultrasound. Further analysis showed that graphene nanoplatelets obtained under higher ultrasound power had a better quality and a lower deformation degree. The second stage of the experiment was to determine the optimal duration of ultrasound operation and the impact of the temperature on the process’s effectiveness. The XRD results showed that only 15 min of pulsed ultrasound operation allowed obtaining high-quality graphene nanoplatelets (Figure S6). A further increase in the duration resulted in a negligible change in the graphene content in the product. Next, the impact of the composition of the solution was checked, in which the suspension obtained during electrolysis was subjected to ultrasound on the structure of the final product. For the test, three compositions were used: pure water, and 33% and 50% mixtures of acetone and water. The obtained suspension was washed using distilled water, added to the different solutions, and then subjected to 630 W ultrasound for 15 min. The analysis of the XRD diffractograms showed that the graphene content in the obtained product was highest for the solution containing 33% acetone (Figure S7).

### 3.2. TEM and SEM Analysis

**Figure 3:**
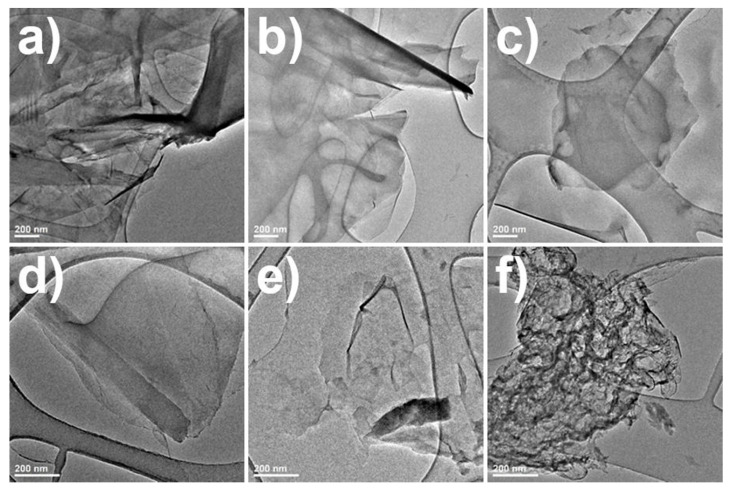
TEM images of graphene samples obtained with optimized synthesis parameters (**a**) with a 10 V voltage, (**b**) after 60 min of electrochemical process, (**c**) in the solution chilled to 5 °C, (**d**) in a solution with an alkaline pH = 10.3, (**e**) after sonification with 630 W ultrasound, and (**f**) after sonification in a solution of water and acetone (33% acetone).

Analysis of the images taken with a transmission electron microscope (TEM) allowed for an assessment of the obtained graphene nanoplatelet size and quality and the presence of byproducts that formed during the synthesis (e.g., carbon dots and graphite particles). The in-depth analysis of the TEM images showed that the size distribution graphene of flakes was quite wide and depended on the synthesis conditions (Figure 3). The analysis of TEM images of graphene formed in an alkaline environment shows that the average size of the obtained flakes was within the range of 1000 to 1200 nm, which was a much higher value as compared to samples obtained in an acidic environment. The number of layers of the obtained graphene in most cases did not exceed 3–5. The graphene flakes obtained at 630 W ultrasound and low temperature had a size in the range of 1600 to 2000 nm. One of the benefits of lowering the temperature was a higher quality of graphene flakes, as the number of layers did not exceed 3 in this case. The TEM images of graphene formed with the use of a 33% acetone solution electrolyte showed that the average size of the flakes obtained was between 1600 and 2000 nm. Additionally, a high level of deformation was observed for the graphene flaked obtained under these conditions. This may have been due to the method of obtaining dry flakes, which are obtained by freeze drying. Since the mixture of acetone and water has a much lower freezing point during drying, the solvent does not evaporate evenly, causing deformation of the flakes.

**Figure 4:**
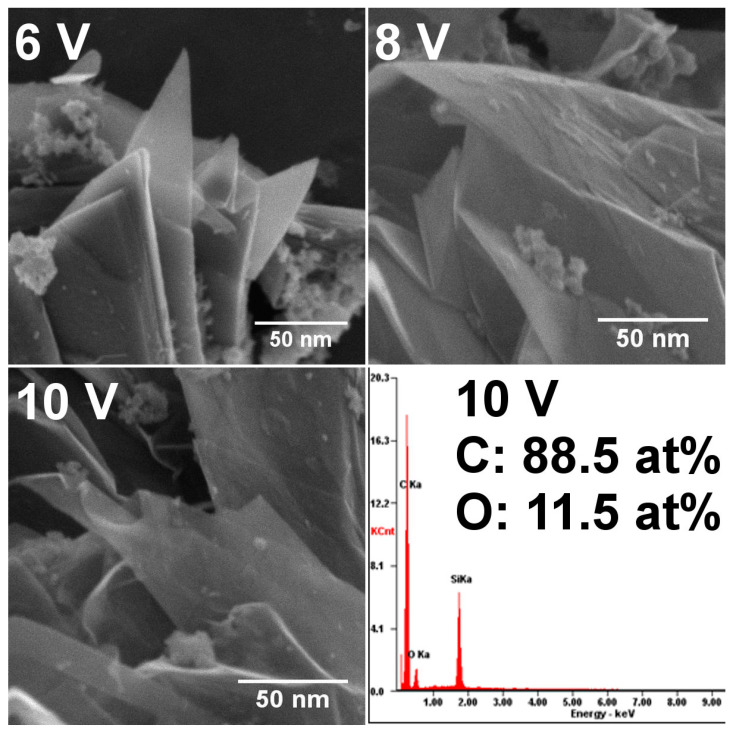
SEM images of graphene samples obtained at different voltage and EDS analysis of the GNPs obtained at 10 V.

The morphology of the samples obtained at different voltage was checked using scanning electron microscopy (Figure 4). The analysis showed that, at lower potential, there still existed thick layers of non-exfoliated graphite. After increasing the voltage in the electrochemical process, the graphite expanded, and graphene flakes with a thickness of 3–5 nm were observed. Because the process involved ions that could oxidize graphene, the oxygen content in the sample with the best quality (10 V) was also checked. For the selected sample, the carbon-to-oxygen ratio was 7.7, which is comparable to the values obtained by Selverda et al. [40] or Liu et al. [41].

### 3.3. Raman Analysis

Raman spectroscopy was used for the qualitative analysis of the obtained graphene flakes. The Raman spectra of graphene were characterized by the presence of three primary bands denoted as D, G, and 2D. Their positions were at about 1350 cm^−1^, 1580 cm^−1^, and 2680 cm^−1^, respectively. The presence of the G band is related to transverse vibrations, while D and 2D bands are related to longitudinal vibrations in the graphene plane. The difference between them consists of the conservation of momentum of the reflected photon. In the case of the D band, momentum is lost due to encountered defects; in the case of the 2D band, the defects are not involved. The analysis of both types of vibrations by comparing the ratio of the respective bands (D to G band) allowed determining the degree of deformation of the obtained carbon structure. By analyzing the full width at half maximum (FWHM) of the 2D band, the number of carbon layers could be determined [42]. The dependence of FWHM on the number of graphene layers is shown in Table 2. The parameters presented are based on experimental results obtained by Hao et al. [42].

The degree of deformation (*D*) of the obtained graphene flakes, as the ratio of the *D*-band intensity to the *G*-band intensity, was determined for the analyzed samples.

(2) $$D=\frac{I_{D}}{I_{G}}.$$

**Figure 5:**
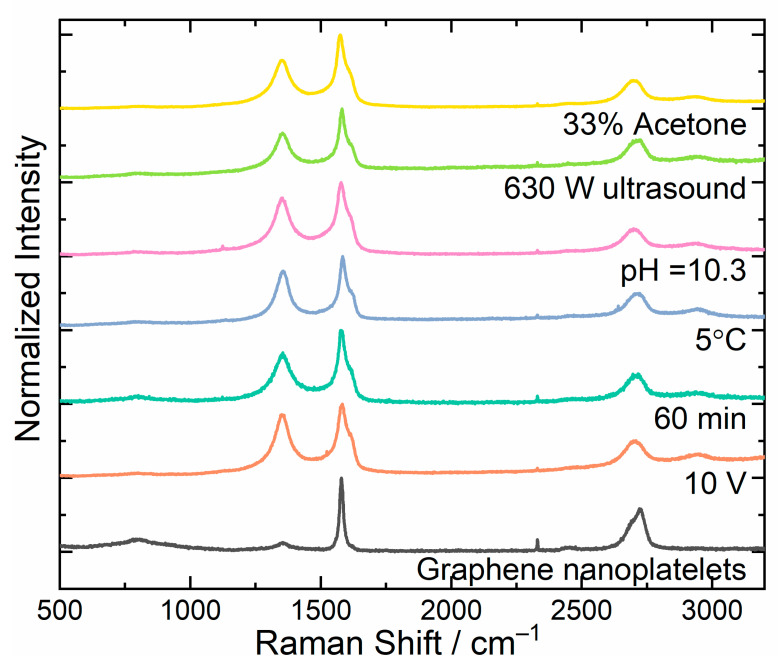
The Raman spectra of selected samples from optimization series.

The Raman spectra of the samples prepared under different conditions, as well as the reference sample, are presented in Figure 5. The Raman spectra of samples withing the whole range of parameters, as well as their FWHM and degree of deformation, are presented in Supplementary Figures S8–S13. For the reference sample, the FWHM of the 2D band was 68.92 cm^−1^, whereas the value of the *D* parameter was 0.1278. This sample was used as a reference to investigate the influence of different process parameters on the quality of the graphene samples.

While determining the optimal voltage of the electrolytic system, the analysis of Raman spectra showed that the sample obtained with a 6 V voltage contained five-layer thick graphene with the lowest degree of deformation, for which *D* = 0.785. Increasing the voltage between the electrodes during the process increased both the degree of deformation and the half-width parameter values (Figure S8). The voltage also had a significant effect on the efficiency of the graphene formation process. It was most efficient when voltage was set to 10 V; in this case, the formation speed was 1.54 g/h, whereas, in the case of 6 V, it was 0.61 g/h. The 12 V voltage caused uneven wear of the electrode and its rapid disintegration. Due to the favorable ratio of quantity to quality, it was decided to continue the research with the voltage of 10 V. During the determination of the optimal duration of electrolysis, the Raman spectra for samples with the electrolysis duration of 45, 60, and 90 min were measured (Figure S9). The results showed that the quality of produced graphene flakes was constant up to a process time of 60 min. Above this time, a significant widening of the half-width of the 2D band could be observed, as well as an increase in the value of the *D* parameter up to 0.851. Another studied parameter affecting the quality of the obtained graphene flakes was the electrolyte temperature. The experiment was conducted in three different ways. In the first one, the temperature was not controlled; after about 5 min of the process, the temperature was measured as 25 °C. In the next variant, the electrolyte was cooled by immersing the reaction container in a vessel with water. The temperature was then set at 15 °C. In the last system. ice was added to the water, and, in this case, the process temperature reached 5 °C. The lowest degree of deformation, as well as the lowest value of the half-width of the 2D band, was obtained for the process carried out at the lowest temperature (Figure S10). Higher temperature caused a faster process and exfoliation of larger flakes with higher deformation degrees. The effect of electrolyte pH on the Raman spectra is shown in Figure S11. The pH of the starting electrolyte was 5.3 and was modified by the addition of ammonia water (increase of pH) or sulfuric acid (decrease of pH). It can be seen that, for the lowest pH (3.2), the graphene flakes were not formed. Additionally, the degree of deformation was lower for a higher pH of the electrolyte. The increase in the degree of deformation with decreasing pH could be explained by the tendency to form graphene oxide (GO) in an acidic environment. The functional groups attached to the monolayers deformed their surfaces, which increased the deformation degree. On the basis of the analysis, it was concluded that the best quality of graphene nanoplatelets was achieved at pH = 10.3. Another of the studied parameters affecting the quality of the obtained graphene flakes was the exposure of the material obtained in the electrolysis process to ultrasound. Ultrasound is intended to promote the delamination of graphene layers. The process was carried out for 15 min at 230 and 630 W. The Raman spectra shown in Figure S12 indicate that both the half-width and the deformation degree parameters were lower for the ultrasound with higher power. The effect of solvent used for sonification on the formation of graphene flakes was checked using water and mixtures of water and acetone in ratios of 2:1 and 1:1. Figure S13 shows that the flakes obtained in the mixture of water and 33% of acetone had the lowest degree of degradation. This shows that the water/acetone (2:1) mixture had an optimal dispersion force and surface tension minimizing the exfoliation energy of the material.

### 3.4. IR Analysis

**Figure 6:**
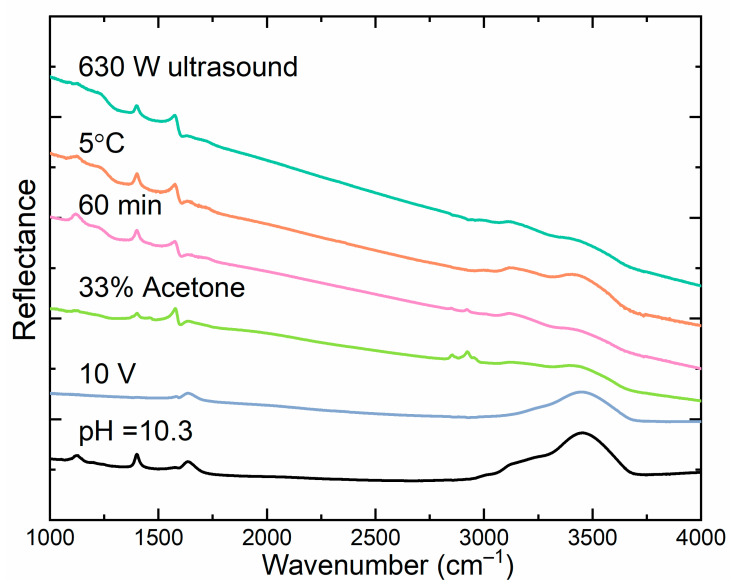
The IR spectra of selected samples obtained under different conditions.

Because of the high scattering of light in the measured samples, graphene was ground with KBr and then formed into a pellet; thus, the results obtained in the IR analysis were evaluated qualitatively rather than quantitatively (Figure 6). Interferences can be seen in some areas of the spectra due to the weak absorption and strong increase in background. Bands exceeding 3000 cm^−1^ were caused by υOH or υNH stretching vibrations of groups attached to graphene, as well as water absorbed by KBr. Bands observed slightly below 3000 cm^−1^ may have arisen due to the presence of aliphatic groups (υCH). The deformational vibrations of δOH and δNH contributed to the band observed in the range of 1500–1750 cm^−1^. The band slightly below 1500 cm^−1^ derived from υC–OH deformational vibrations and δCH deformation vibrations. The bands observed about 1120 cm^−1^ could be attributed to υCO and/or υCN stretching vibrations.

The changes in IR spectra depending on the method used were small, but allowed some very general conclusions to be drawn. The sample that was not modified (10 V) contained the fewest types or no functional groups on the surface, as the observed bands above 1600 and 3000 cm^−1^ could have arisen from the hygroscopicity of KBr. Any of the other modifications resulted in the formation of the same bands below 1500 cm^−1^, indicating the appearance of similar amounts of characteristic functional groups. The use of organic modifiers, such as acetone, resulted in characteristic absorption near 3000 cm^−1^, suggesting the presence of CH groups.

### 3.5. Zeta Potential

The zeta potential is a measurement of the repulsive and attraction forces between particles suspended in liquids. The investigation was performed on selected samples from previous experiments. For the experiment, freeze-dried flakes were suspended in water using ultrasound. It is known that a lower zeta potential value indicates a higher stability of the particle dispersion in solution. Stable colloids are formed when the absolute value of potential is higher than +30 or lower than −30 mV [43]. The values of measured zeta potential are shown in Table 3 and presented in Figure S14. The results showed that the additional factors such as temperature, pH, ultrasound and addition of acetone improved dispersion in the solution, indicating the presence of additional groups on the surface of the graphene flakes additional groups. The lowest potential value was achieved for the sample obtained in a mixture of 33% acetone with a value of −42.2 mV.

## 4. Discussion

In order to optimize the process of obtaining graphene nanoplatelets by combining the electrochemical exfoliation and exfoliation methods in liquid phase using ultrasound, the following parameters were investigated: applied voltage, process duration, temperature, and pH of the electrolyte. The influence of acetone content in the solution of graphene obtained after electrolytic exfoliation and interaction of this solution with different power and duration of ultrasound was also examined. By selecting the optimal voltage (10 V), it was possible to obtain a significantly larger mass of the product while also providing a slight difference in its quality. Another important factor that increased the yield of the process was the temperature of the reaction environment. At 5 °C (ice bath), the mass of the graphene nanoplatelets could even be even doubled as compared to the exfoliation in 15 °C (water bath) and tripled if the temperature was not controlled. A change in the electrolyte pH had a positive effect on the quality of the obtained graphene. However, the use of NH_3_·H_2_O would increase the price of the process due to the need of using large amounts of it. Considering the ratio of product quality to the price, it was decided to continue the research without changing the pH of the electrolyte. On the basis of the conducted measurements, it was found that the most favorable power and operating time of ultrasound process were 630 W and 15 min, respectively. It is also recommended to use an ice bath during the operation of ultrasound. Lowering the temperature prevents the separated graphene layers from reassembling, which ensures better quality. Mass analysis showed that, with an increase in acetone concentration in the electrolyte, the amount of the product obtained dropped sharply. The average relative weight of graphene obtained with a 33% acetone solution was halved, while the difference was up to 80% with a 50% solution. The experimental work aimed to obtain the largest possible amount of good-quality product with minimal financial and time consumption. As such, sonification of graphene should be conducted in water or 33% acetone solution. On the basis of the analysis of data obtained with the use of XRD, TEM, Raman/IR spectroscopy, and zeta potential measurements, the following parameters were determined as optimal to give the best quality, highest mass, and the lowest process price of the product: constant voltage of the system equal to 10 V, process temperature reduced by an ice bath, 5.3 pH of the electrolyte (0.1 M solution of (NH_4_)_2_SO_4_), and process time of 60 min. The solution of the electrolysis product should be prepared using pure water (or 33% acetone if a better quality of graphene is needed) and then subjected to ultrasound (630 W) for 15 min. To obtain dry graphene nanoplatelets, the sonicated product should be frozen and evaporated using a freeze-dryer.

## Figures and Tables

**Table 1:** Analyzed factors of the electrochemical exfoliation and exfoliation in liquid-phase procedures.

Synthesis Factor	Unit	
Voltage	V	6, 8, 10, 12
Electrolysis duration	min	45, 60, 90
Temperature during electrolysis	°C	5, 15, 25
pH of the electrolyte	-	3.2, 4.2, 5.3, 6.1, 7.7, 8.7, 9.6, 10.3
Acetone content in the electrolyte	%	0, 33, 50
Ultrasound power	W	230, 630
Ultrasound duration	min	15, 30, 45, 60, 90
Temperature during sonification	°C	5, 20, 30

**Table 2:** Dependence of FWHM on the number of graphene layers.

FWHM [cm^−1^]	Number of Graphene Layers
27.5 ± 3.8	1
51.7 ± 1.7	2
56.2 ± 1.6	3
63.1 ± 1.6	4
66.1 ± 1.4	5

**Table 3:** Zeta potential of obtained graphene flakes suspending in the water.

Sample	Zeta Potential/mV
10 V	−28.9
60 min	−28.3
5 °C	−35.3
pH 10.3	−37.5
630 W ultrasound	−32.3
33% acetone	−42.2

## Data Availability

The data presented in this study are available on request from the corresponding author.

## Supplementary Materials

The following supporting information can be downloaded at https://www.mdpi.com/article/10.3390/ma16062188/s1: Figure S1. The influence of applied voltage in electrochemical process on the structure of obtained graphene samples; Figure S2. The influence of time of electrochemical process on the structure of graphene samples; Figure S3. The influence of temperature of electrochemical process on the structure of obtained graphene samples; Figure S4. The influence of pH of solution in electrochemical process on the structure of obtained graphene samples; Figure S5. The influence of ultrasound power on the structure of obtained graphene samples; Figure S6. The influence of ultrasound time on the structure of obtained graphene samples; Figure S7. The influence of solution composition on the structure of obtained graphene samples; Figure S8. The influence of voltage applied in electrochemical process on Raman spectra, FWHM of 2D peak, and degree of deformation (D) of obtained graphene samples; Figure S9. The influence of time of electrochemical process on Raman spectra, FWHM of 2D peak, and degree of deformation (D) of obtained graphene samples; Figure S10. The influence of temperature of electrochemical process on Raman spectra, FWHM of 2D peak, and degree of deformation (D) of obtained graphene samples; Figure S11. The influence of pH of solution in electrochemical process on Raman spectra, FWHM of 2D peak, and degree of deformation (D) of obtained graphene samples; Figure S12. The influence of ultrasound power on Raman spectra, FWHM of 2D peak, and degree of deformation (D) of obtained graphene samples; Figure S13. The influence of solution composition on Raman spectra, FWHM of 2D peak, and degree of deformation (D) of obtained graphene samples; Figure S14. Zeta potential distribution of graphene nanoplatelets obtained in different process conditions measured for suspension in water.
