# Supplementary material for: Optimization of the Electrochemical Method of Obtaining Graphene Nanoplatelets (GNPs)
Source: Materials (Basel). 2023 Mar 9;16(6):2188. doi: 10.3390/ma16062188 (PMC10057930; doi:10.3390/ma16062188)
Supplement: Supplementary file 1 [file materials-16-02188-s001.zip › materials-2203463-supplementary.pdf]

Supplementary Materials

Optimization of the Electrochemical Method of Obtaining Graphene Nanoplatelets (GNPs)

Adrianna Grabowska [1,2], Jerzy Kowalczyk [2], Robert Tomala [2], Maciej Ptak [2], Małgorzata Małecka [2], Anna Wędzyńska [2], Mariusz Stefanski [2], Wiesław Stręk [2] and Paweł Głuchowski [2,*]

[1] Faculty of Chemistry, Wroclaw University of Science and Technology, 50-370 Wroclaw, Poland; adagrabowska10@gmail.com
[2] Institute of Low Temperature and Structure Research, Polish Academy of Sciences, 50-422 Wroclaw, Poland; jerzy.kowalczyk@pwr.edu.pl (J.K.); r.tomala@intibs.pl (R.T.); m.ptak@intibs.pl (M.P.); m.malecka@intibs.pl (M.M.); a.wedzynska@intibs.pl (A.W.); m.stefanski@intibs.pl (M.S.); w.strek@intibs.pl (W.S.)
* Correspondence: p.gluchowski@intibs.pl

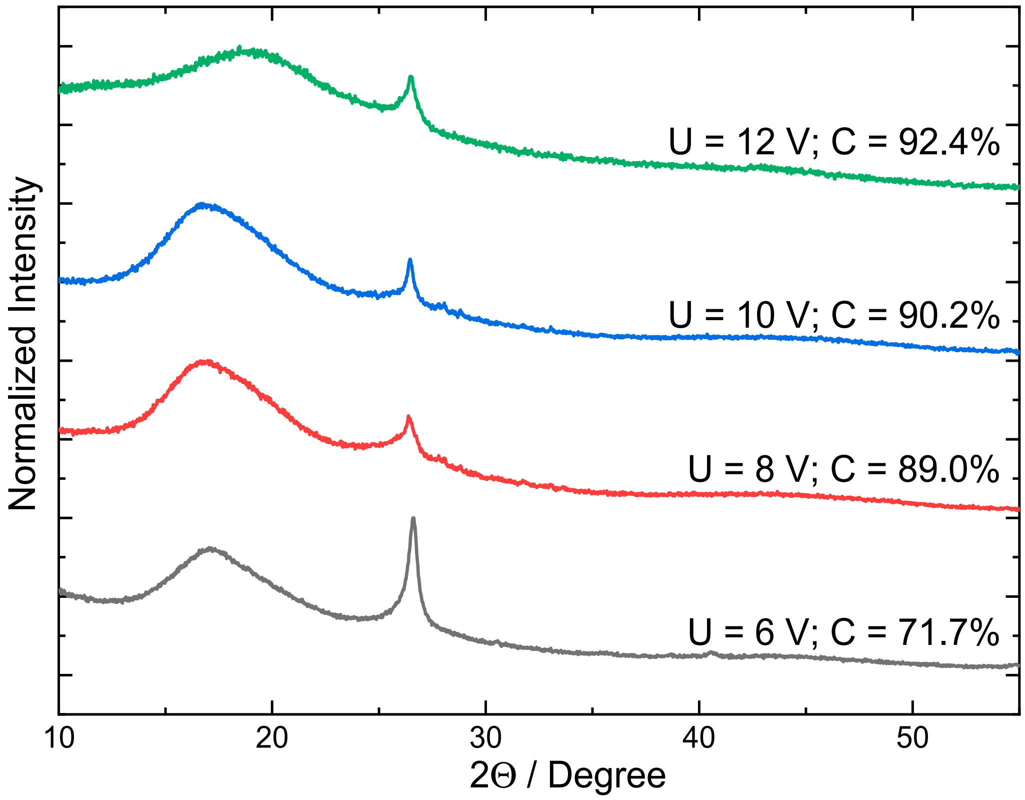

Figure S1. The influence of applied voltage in electrochemical process on the structure of obtained graphene samples.

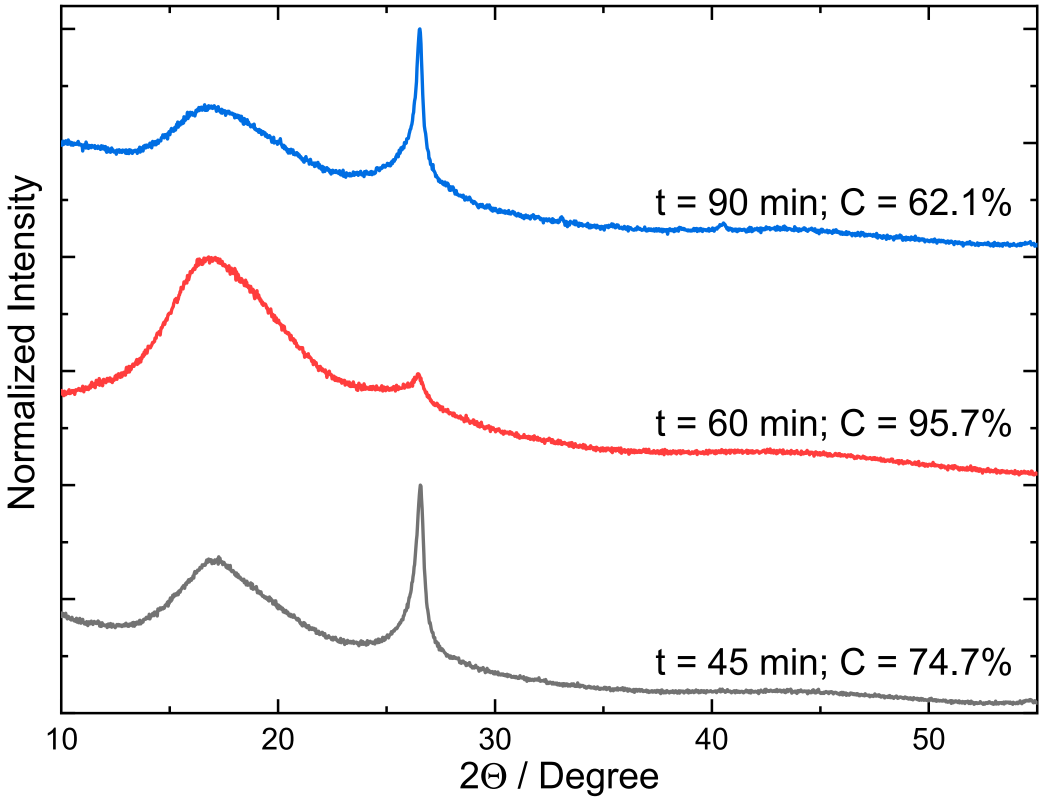

Figure S2. The influence of time of electrochemical process on the structure of graphene samples.

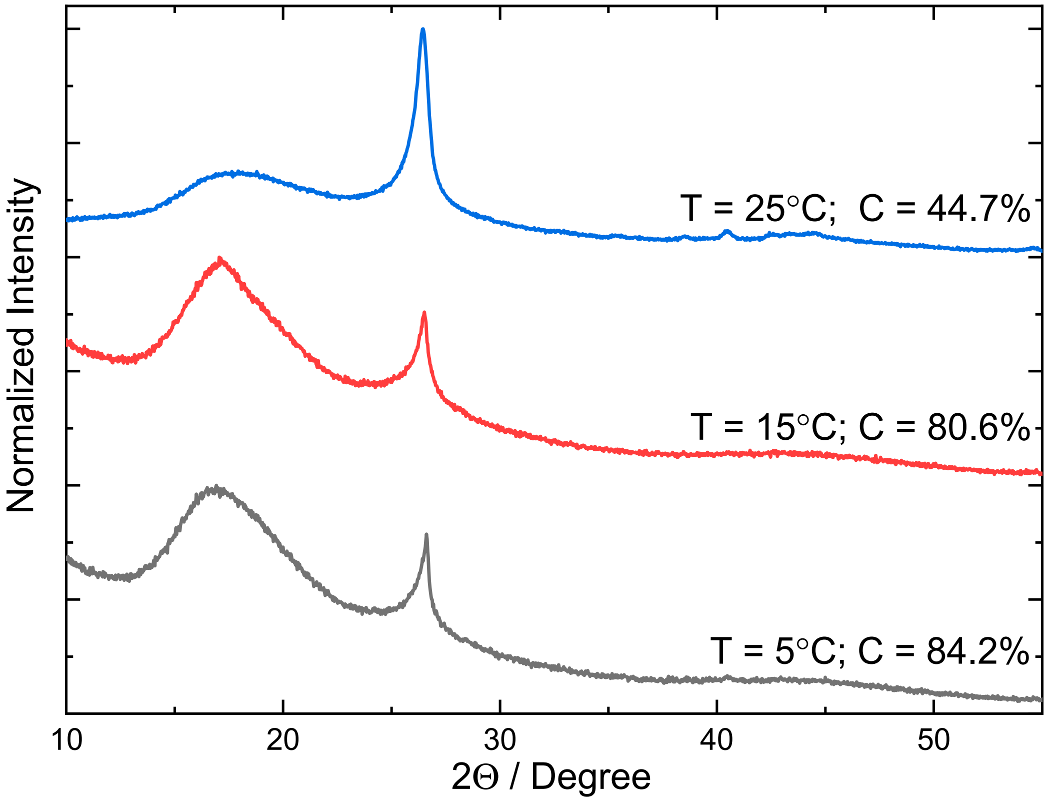

Figure S3. The influence of temperature of electrochemical process on the structure of obtained graphene samples.

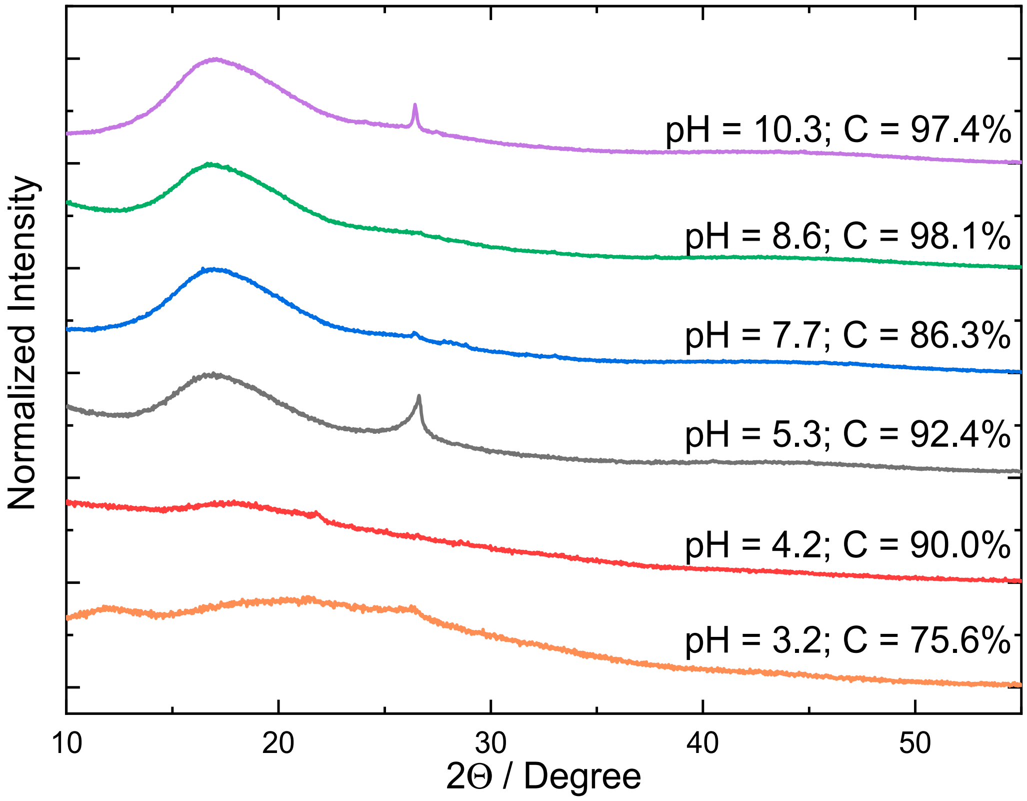

Figure S4. The influence of pH of solution in electrochemical process on the structure of obtained graphene samples.

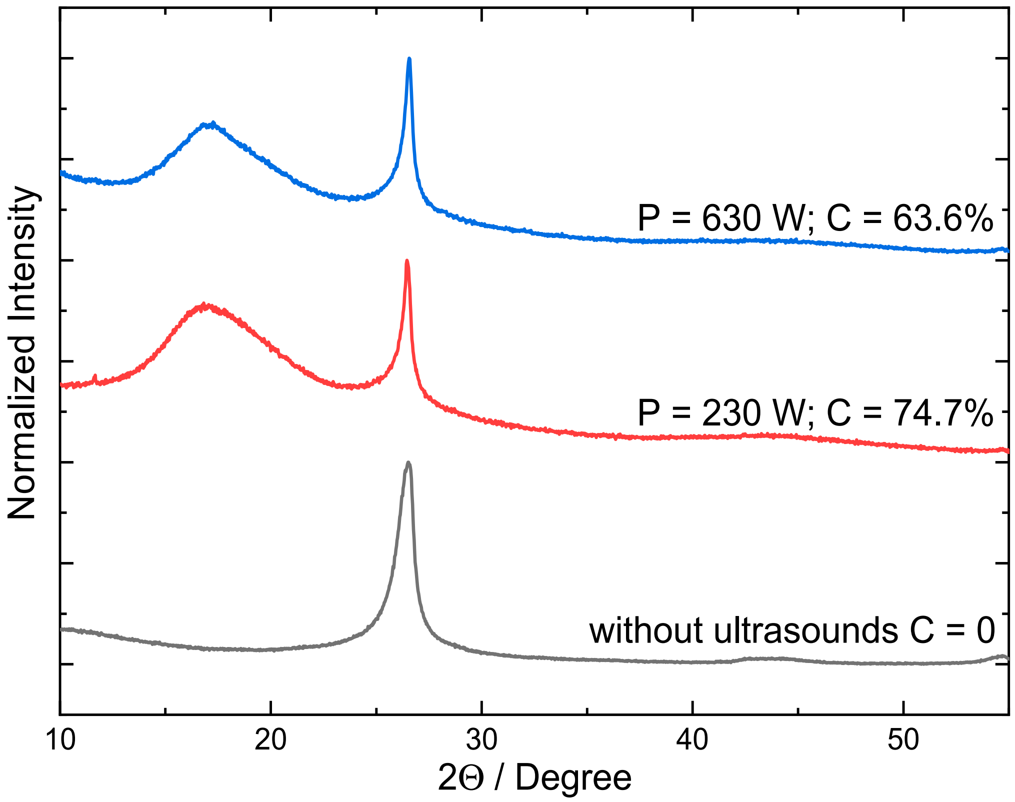

Figure S5. The influence of ultrasounds power on the structure of obtained graphene samples.

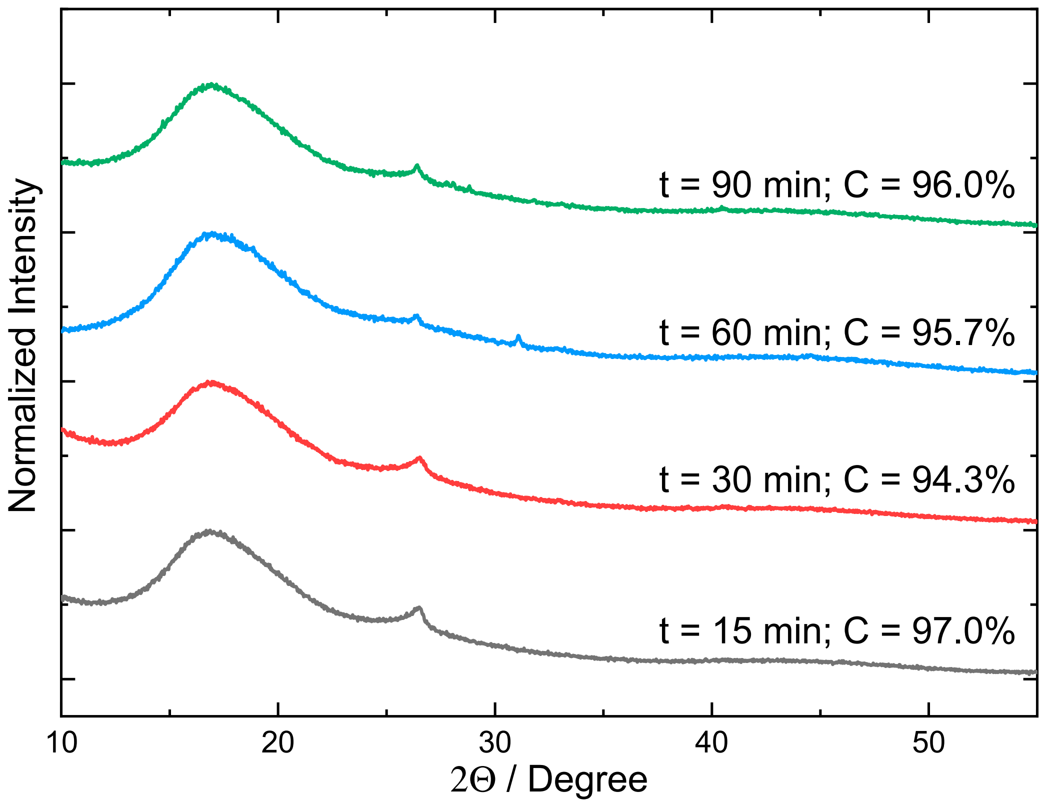

Figure S6. The influence of ultrasounds time on the structure of obtained graphene samples.

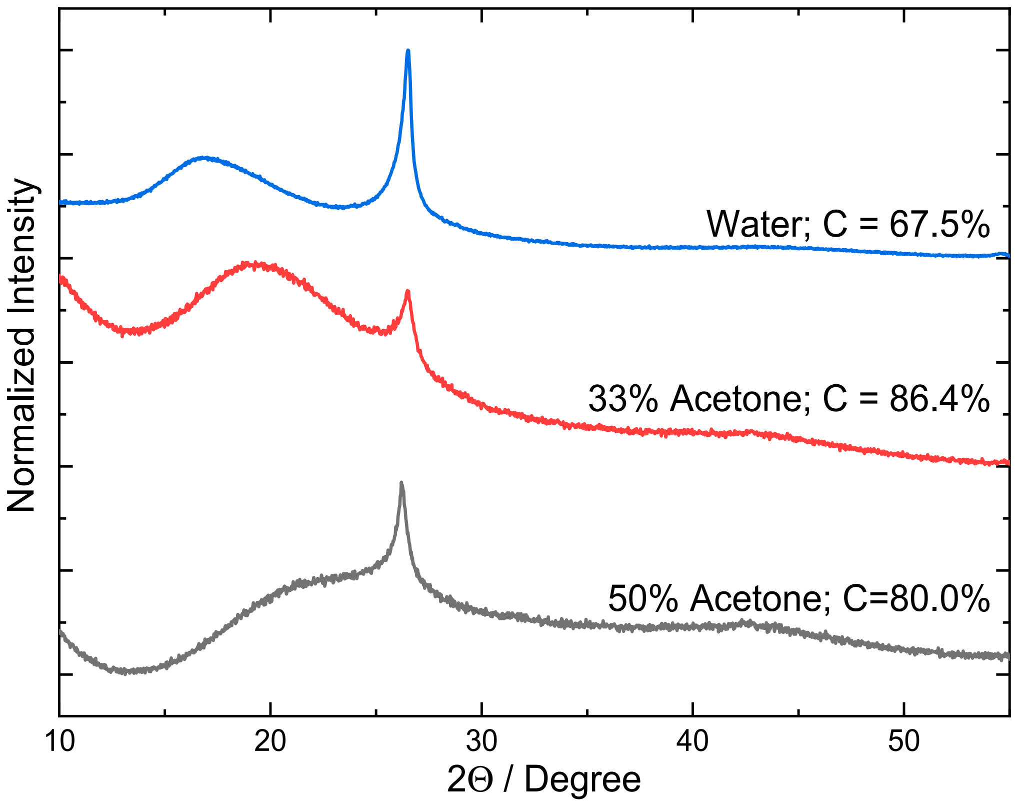

Figure S7. The influence of solution composition on the structure of obtained graphene samples.

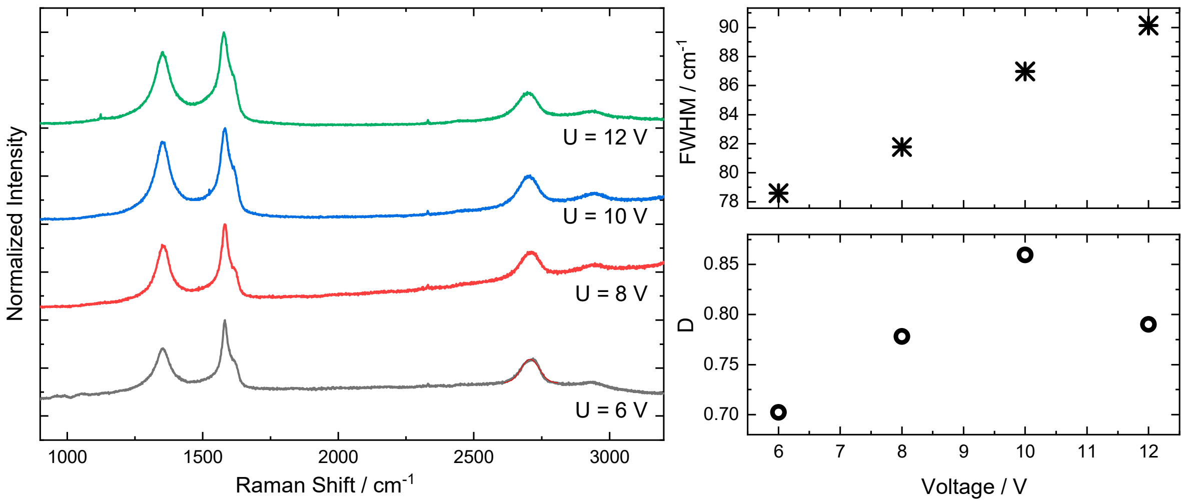

Figure S8. The influence of voltage applied in electrochemical process on Raman spectra, FWHM of 2D peak and degree of deformation (D) of obtained graphene samples.

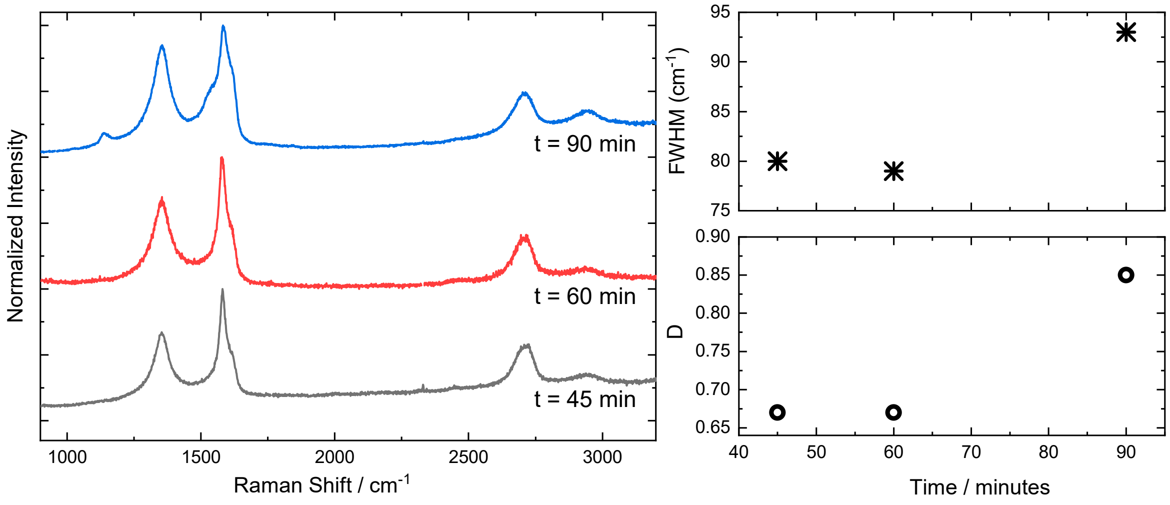

Figure S9. The influence of time of electrochemical process on Raman spectra, FWHM of 2D peak and degree of deformation (D) of obtained graphene samples.

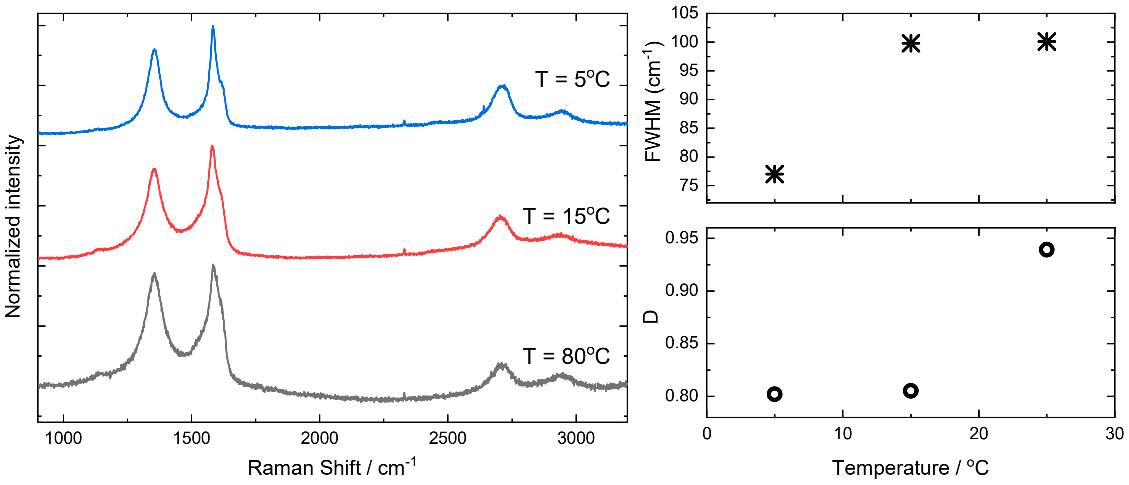

Figure S10. The influence of temperature of electrochemical process on Raman spectra, FWHM of 2D peak and degree of deformation (D) of obtained graphene samples.

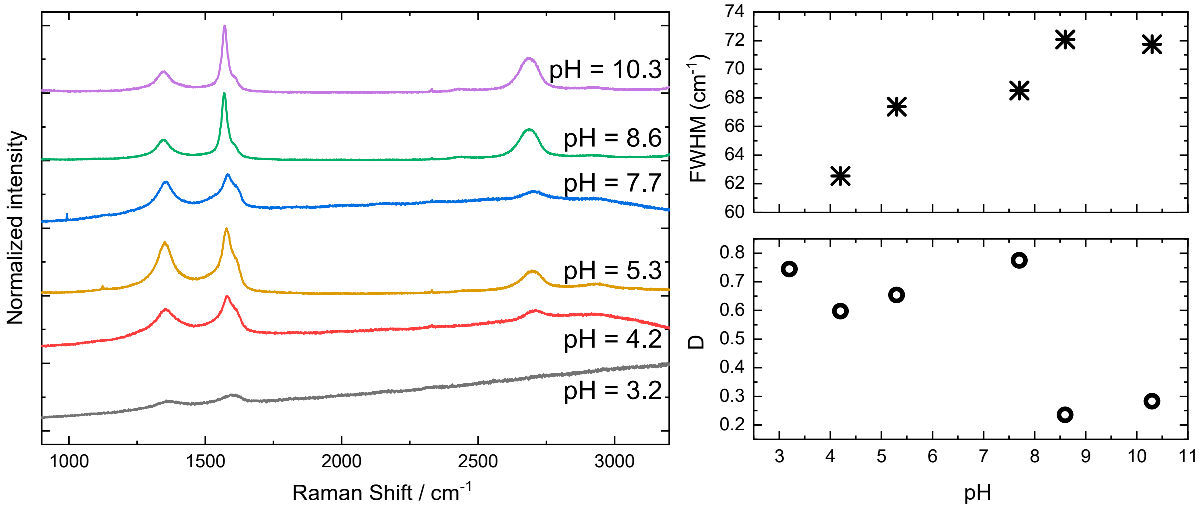

Figure S11. The influence of pH of solution in electrochemical process on Raman spectra, FWHM of 2D peak and degree of deformation (D) of obtained graphene samples.

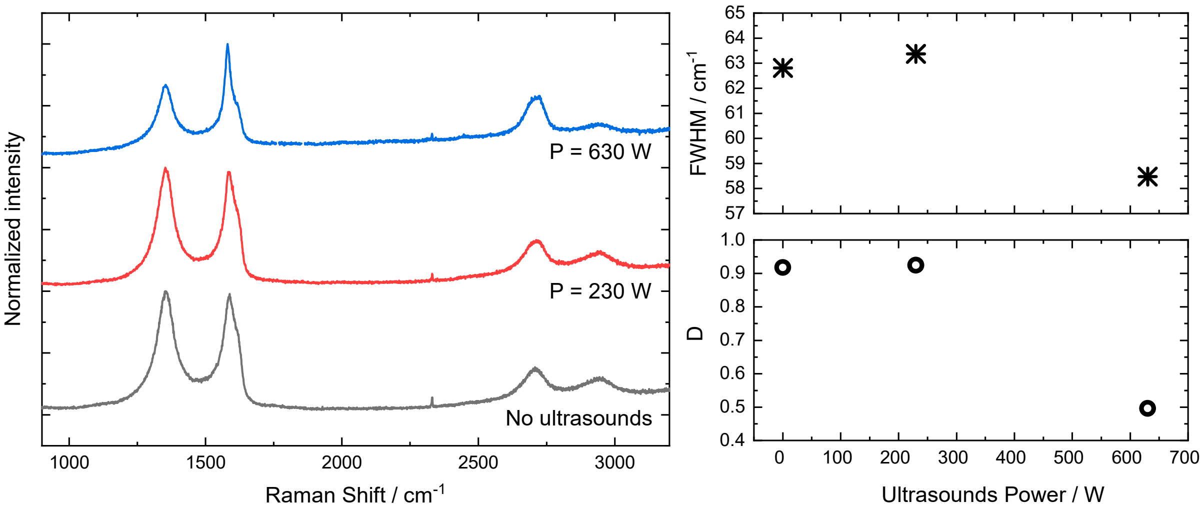

Figure S12. The influence of ultrasound power on Raman spectra, FWHM of 2D peak and degree of deformation (D) of obtained graphene samples.

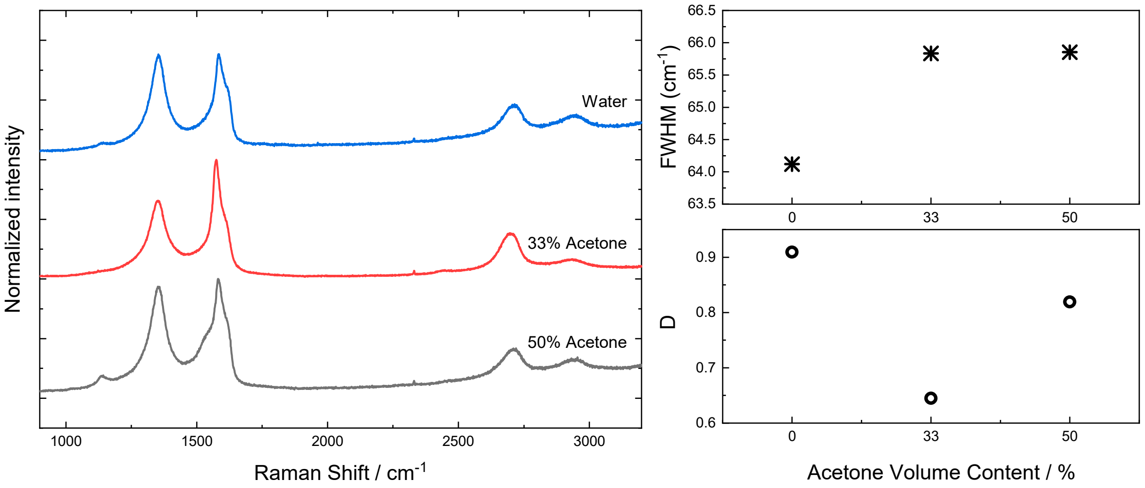

Figure S13. The influence of solution composition on Raman spectra, FWHM of 2D peak and degree of deformation (D) of obtained graphene samples.

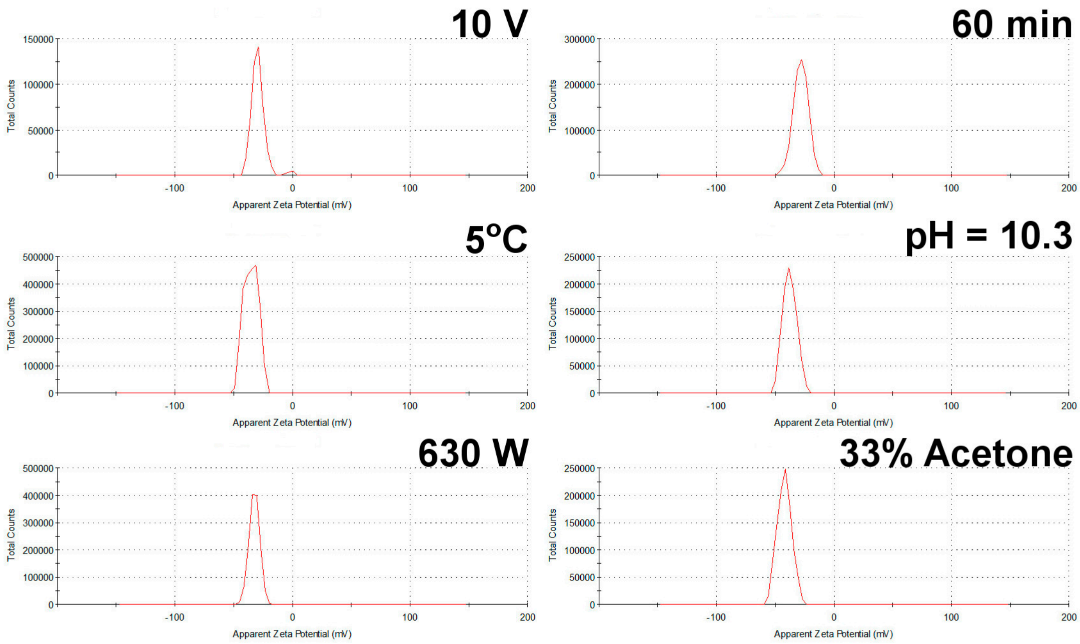

Figure S14. Zeta potential distribution of the graphene nanoplatelets obtained at different process conditions measured for suspension in water.
